# Use of the prognostic nutrition index as a predictive biomarker in small-cell lung cancer patients undergoing immune checkpoint inhibitor treatment in the Chinese alpine region

**DOI:** 10.3389/fonc.2023.1041140

**Published:** 2023-03-15

**Authors:** Yunjiao Wu, Jing Yang, Xinyi Qiao, Yingjie Li, Rui Zhao, Tie Lin, Xiaoli Li, Meng Wang

**Affiliations:** ^1^ Department of Respiratory Medical Oncology, Harbin Medical University Cancer Hospital, Heilongjiang, Harbin, China; ^2^ Chongqing Engineering Research Center for Processing and Storage of Distinct Agricultural Products, Chongqing Technology and Business University, Chongqing, China; ^3^ Department of Surgery, The First Affiliated Hospital of Harbin Medical University, Heilongjiang, Harbin, China

**Keywords:** prognostic nutritional index (PNI), small cell lung cancer (SCLC), PD-L1/PD-1 inhibitors, immune-related adverse events (irAEs), prognostic factor

## Abstract

**Background:**

Whether the prognostic nutritional index (PNI), which is suggested to reflect systemic inflammation and nutritional status of patients, could be used as an effective prognostic factor for small-cell lung cancer (SCLC) has not yet been clarified. The purpose of this study was to verify the prognostic value of the PNI in SCLC patients treated with programmed cell death ligand-1/programmed cell death 1 (PD-L1/PD-1) inhibitors in the alpine region of China.

**Methods:**

SCLC patients treated with PD-L1/PD-1 inhibitors monotherapy or combined with chemotherapy between March 2017 and May 2020 were included. Based on the values of serum albumin and total lymphocyte count, the study population was divided into two groups: high and low PNI. The Kaplan-Meier method was used to compute the median survival time and the log-rank test was used to compare the two groups. To evaluate the prognostic value of the PNI, univariable and multivariable analyses of progression-free survival (PFS) and overall survival (OS) were performed. The correlations between PNI and DCR or ORR were calculated by Point biserial correlation analysis.

**Results:**

One hundred and forty patients were included in this study, of which, 60.0% were high PNI (PNI > 49.43) and 40.0% were low PNI (PNI ≤ 49.43). Results indicated that the high PNI group had better PFS and OS than the low PNI group in the patients who received PD-L1/PD-1 inhibitors monotherapy (median PFS: 11.0 vs. 4.8 months, *p* < 0.001 and median OS: 18.5 vs. 11.0 months, *p* = 0.004). Similarly, better PFS and OS were associated with an increase in PNI level in the patients who accepted PD-L1/PD-1 inhibitors combined with chemotherapy (median PFS: 11.0 vs. 5.3 months, *p* < 0.001 and median OS: 17.9 vs. 12.6 months, *p* = 0.005). Multivariate Cox-regression model showed that high PNI was significantly related to better PFS and OS in patients who accepted PD-L1/PD-1 inhibitors monotherapy or combined with chemotherapy (PD-L1/PD-1 inhibitors monotherapy: PFS: HR = 0.23, 95% CI: 0.10–0.52, *p* < 0.001 and OS: HR = 0.13, 95% CI: 0.03–0.55, *p =* 0.006; PD-L1/PD-1 inhibitors combined with chemotherapy: PFS: HR = 0.34, 95% CI: 0.19–0.61, *p* < 0.001 and OS: HR = 0.53, 95% CI: 0.29–0.97, *p =* 0.040, respectively). Additionally, Point biserial correlation analysis between PNI and disease control rate (DCR) showed that PNI status was positively correlated with DCR in SCLC patients receiving PD-L1/PD-1 inhibitors or combined with chemotherapy (r = 0.351, *p* < 0.001; r = 0.285, *p* < 0.001, respectively).

**Concussions:**

PNI may be a promising biomarker of treatment efficacy and prognosis in SCLC patients treated with PD-L1/PD-1 inhibitors in the alpine region of China.

## Introduction

Lung cancer continues to be the leading cause of cancer-related deaths worldwide, particularly in the alpine region of China. Small-cell lung cancer (SCLC) accounts for 15% of all lung cancers and has a 5-year survival of 1–2%, as most patients present with late-stage disease (1–4). Immune checkpoint inhibitors (ICIs) such as programmed cell death ligand-1/programmed cell death 1 (PD-L1/PD-1) inhibitors have revolutionized the therapeutic paradigm of SCLC. Gay et al. found that molecular subtypes classified as “SCLC-I” derived a significant overall survival (OS) benefit from immune checkpoint blockade (HR = 0.57, 95% CI: 0.32–1.00), suggesting patients with advanced SCLC may benefit from ICIs (5). Nevertheless, the absolute improvements in progression free survival (PFS) and OS are not occur to all SCLC patients. Therefore, there is an urgent need to determine an appropriate biomarker to identify which SCLC patients may benefit from PD-L1/PD-1 inhibitor treatment (6, 7).

Related studies have shown that PD-L1 expression is low or absent in SCLC patients (8–11). Recently, Iams et al. has demonstrated that even PD-L1 negative patients could responded well to inhibitor treatment (12). Therefore, PD-L1 expression is not used as a predictive biomarker in SCLC patients receiving PD-L1/PD-1 inhibitor treatment. Tumor mutational burden (TMB) has been demonstrated to be related to the efficacy of PD-L1/PD-1 inhibitor treatment in several large clinical trials (13, 14). However, the TMB does not have a clear cut-off value. Therefore, further studies are needed to generate a consensus on utilizing the TMB in clinical practice. Additionally, microsatellite instability (MSI) and tumor-infiltrating lymphocytes (TILs) are promising predictive biomarkers of ICIs response that warrant further evaluation (15, 16). Currently, PD-L1/PD-1 inhibitor treatment in SCLC patients lacks robust indicators for determining which patients will benefit.

Typically, obtaining tumor specimens during the process of treatment is difficult. Therefore, a non-invasive biomarker that can conveniently predict the efficacy and prognosis of immunotherapy is needed. The progression of SCLC is closely associated with systemic inflammation and nutritional status. Several studies have shown that inflammatory and nutritional peripheral blood parameters may be potential biomarkers of the effects of immunotherapy outcomes in patients with melanoma and head and neck squamous cell carcinoma (HNSCC) (17–19).

Prognostic nutritional index (PNI) is obtained by the level of serum albumin and peripheral lymphocytes and is proposed to assess immune-nutritional status (20). A few studies have shown that low PNI status is associated with an unfavorable prognosis in gastrointestinal and colorectal cancer (21, 22).

Owing to the roles played by the PNI, we hypothesized that there is a relationship between treatment response and the PNI in SCLC patients. The aim of the present study was to investigate whether pretreatment PNI is a predictive biomarker in SCLC patients undergoing PD1/PD-L1 treatment in the Chinese Alpine Region.

## Patients and methods

### Patients selection

We retrospectively enrolled 140 SCLC patients undergoing PD-L1/PD-1 inhibitor treatment at the Harbin Medical University Cancer Hospital between March 2017 and May 2020. The inclusion criteria for SCLC patients treated with PD-L1/PD-1 inhibitors were as follows (1): patients who were diagnosed with SCLC by histopathology or cytopathology; (2) Eastern Cooperative Oncology Group performance status (ECOG PS) ≤ 2 points; (3) at least two cycles of PD-L1/PD-1 inhibitor therapy, PD-L1/PD-1 inhibitors combined with chemotherapy or PD-L1/PD-1 inhibitors as monotherapy. The exclusion criteria for SCLC patients treated with PD-L1/PD-1 inhibitors were as follows: (1) lack of complete clinicopathological information or laboratory data before PD-L1/PD-1 inhibitor treatment; (2) patients with malignancy in other organs or hematological diseases, autoimmune diseases, and systemic immunosuppression. Basic clinical and pathological data were collected from patients who met these criteria. This study was approved by the Institutional Review Board of the Harbin Medical University Cancer Hospital.

### Data collection and definitions

Primary laboratory data from before PD-L1/PD-1 inhibitor treatment and clinicopathologic data were retrieved from SCLC patient medical records. The PNI was calculated as 10 × serum albumin (g/dl) + 0.005 × total lymphocyte count (per mm^3^) (20).

### Evaluation

Two doctors independently evaluated drug effectiveness based on image examinations every 8–12 weeks, according to the Response Evaluation Criteria in Solid Tumors guidelines version 1.1 (RECIST1.1). Immune-related adverse events (irAEs) were assessed by two doctors independently according to the Common Terminology Criteria for Adverse Events (CTCAE) of the National Cancer Institute (version 4.03). The disease control rate (DCR) was defined as complete plus partial response plus stable disease, and the overall response rate (ORR) was defined as complete plus partial response. OS was defined as the time from the start of treatment with PD-L1/PD-1 inhibitors to death. PFS was defined as the time from the start of treatment with PD-L1/PD-1 inhibitors to disease progression.

### Statistical analyses

Statistical analyses were performed using IBM SPSS Statistic 25.0. The clinicopathological data of SCLC patients was compared by Chi-squared or Fisher’s exact test. Continuous variables were compared using Student’s *t* test. We generated ROC curves and calculated the optimal cut-off values for PNI, lymphocytes and albumin. The Kaplan-Meier method and log-rank test were used to perform survival analyses. Univariate and multivariate Cox regression analyses were used to identify independent prognostic factors associated with OS and PFS. The correlations between PNI and DCR or ORR were calculated by Point biserial correlation analysis. The impact of the PNI on DCR and ORR is represented by a column diagram. A *p* value < 0.05 was considered statistically significant.

## Results

### Patient clinical characteristics and outcomes

A total of 140 patients with SCLC treated with PD-L1/PD-1 inhibitors were identified for our analysis. Among these, 60.0% (n = 84) of the patients had high PNI and 40.0% (n = 56) had low PNI. Further, 43.6% (n = 61) of the patients received PD-1 inhibitor treatment and 56.4% (n = 79) received PD-L1 inhibitor treatment. Of all the patients that received PD-L1/PD-1 inhibitor therapy (n = 140), 43.6% (n = 61) received PD-L1/PD-1 inhibitors monotherapy. In addition, ECOG PS was 0–1 in 80.7% (n = 113), and 2 in 19.3% (n = 27) of patients. Moreover, 22.1% (n = 31) of patients had brain metastases, 31.4% (n = 44) had liver metastases, 32.9% (n = 46) had bone metastases, 38.6% (n = 54) had pleural or pericardial metastases, and 11.4% (n = 16) had adrenal metastases. Characteristics for the entire cohort and different PNI groups are summarized in **Table 1**.

**Table 1:** Clinical characteristics of the 140 patients received PD-L1/PD-1 inhibitors treatment.

Clinical characteristics	Total [n (%)]	High PNI [n (%)]	Low PNI [n (%)]	*p* value
Total	140 (100)	84 (60.0)	56 (40.0)	
Age				0.015
≤60	60 (42.9)	43 (51.2)	17 (30.4)	
> 60	80 (57.1)	41 (48.8)	39 (69.6)	
Gender				0.032
Male	73 (52.1)	50 (59.5)	23 (41.1)	
Female	67 (47.9)	34 (40.5)	33 (58.9)	
Smoking history				0.227
Yes	112 (80.0)	70 (83.3)	42 (75.0)	
No	28 (20.0)	14 (16.7)	14 (25.0)	
ECOG PS				0.007
0-1	113 (80.7)	74 (88.1)	39 (69.6)	
2	27 (19.3)	10 (11.9)	17 (30.4)	
Stage				
Extended stage	125 (89.3)	72 (85.7)	53 (94.6)	0.094
Limited stage	15 (10.7)	12 (14.3)	3 (5.4)	
Therapy line				0.269
1	68 (48.6)	44 (52.4)	24 (42.9)	
≥2	72 (51.4)	40 (47.6)	32 (57.1)	
Immunotherapy drug				0.210
PD-1	61 (43.6)	33 (39.3)	28 (50.0)	
PD-L1	79 (56.4)	51 (60.7)	28 (50.0)	
Regimen				0.003
Combination therapy	79 (56.4)	56 (66.7)	23 (41.1)	
Monotherapy	61 (43.6)	28 (33.3)	33 (58.9)	
Brain metastases				0.561
Yes	31 (22.1)	20 (23.8)	11 (19.6)	
No	109 (77.9)	64 (76.2)	45 (80.4)	
Liver metastases				0.372
Yes	44 (31.4)	24 (28.6)	20 (35.7)	
No	96 (68.6)	60 (71.4)	36 (64.3)	
Bone metastases				0.883
Yes	46 (32.9)	28 (33.3)	18 (32.1)	
No	94 (67.1)	56 (66.7)	38 (67.9)	
Pleural or pericardial metastases				0.009
Yes	54 (38.6)	25 (29.8)	29 (51.8)	
No	86 (61.4)	59 (70.2)	27 (48.2)	
Adrenal metastases				0.159
Yes	16 (11.4)	7 (8.3)	9 (16.1)	
No	124 (88.6)	77 (91.7)	47 (83.9)	
IrAEs				
Yes	52 (37.1)	38 (45.2)	14 (25.0)	0.015
No	88 (62.9)	46 (54.8)	42 (75.0)	

PD-L1/PD-1, programmed death-ligand 1/programmed death-1; PNI, prognostic nutritional index; ECOG-PS, Eastern Cooperative Oncology Group Performance Status; irAEs, immune-related adverse events.

### Identification of PNI, albumin, and lymphocyte cut-off values

**Figure 1:**
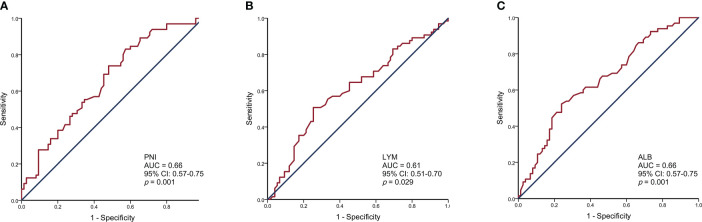
Receiver operating characteristic curve analysis for the prognostic nutritional index **(A)**, lymphocytes **(B)**, and albumin **(C)** to predict the prognosis of SCLC patients.

The PNI, lymphocyte, and albumin values in SCLC patients ranged from 35.15 to 65.10, 0.54 to 2.91, and 30.90 to 55.00, respectively. We generated ROC curves and calculated the optimal cut-off values for PNI, lymphocytes, and albumin (**Figure 1**). The optimal cut-off values for PNI, lymphocytes, and albumin were 49.43, 1.93, and 43.40, respectively. Based on the optimal cut-off values, we divided patients into two groups for further analysis: Low PNI (PNI ≤ 49.43) and High PNI (PNI > 49.43), Low Lymphocyte (LYM) (LYM ≤ 1.93) and High LYM (LYM > 1.93), or Low Albumin (ALB) (ALB ≤ 43.40) and High ALB (ALB > 43.40).

### Association between the PNI and the prognostic utility of SCLC patients received PD-L1/PD-1 inhibitors treatment

**Figure 2:**
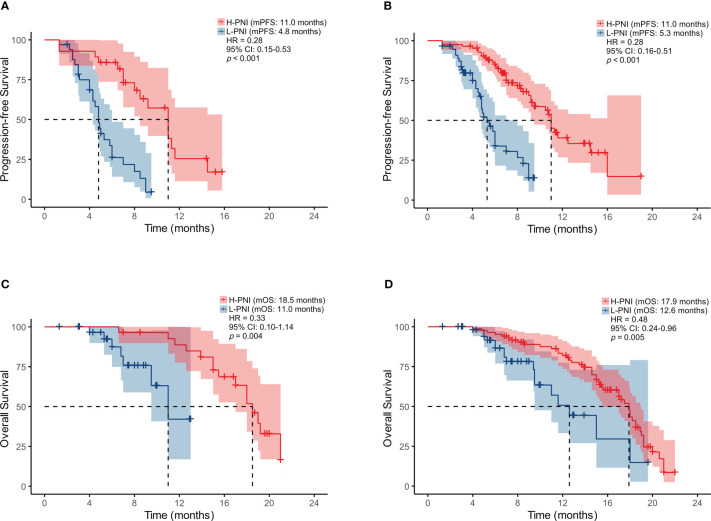
Kaplan-Meier survival curves displaying PFS according to PNI group in SCLC patients treated with **(A)** PD-L1/PD-1 inhibitors monotherapy or **(B)** combined with chemotherapy; OS by PNI group in SCLC patients treated with **(C)** PD-L1/PD-1 inhibitors monotherapy or **(D)** combined with chemotherapy.

As shown in **Figure 2**, a high PNI was associated with better PFS in the patients who accepted PD-L1/PD-1 inhibitors monotherapy or combined with chemotherapy (median PFS: 11.0 vs. 4.8 months, HR = 0.28, *p* < 0.001 and median PFS: 11.0 vs. 5.3 months, HR = 0.28, *p* < 0.001) (**Figures 2A, B**). Similarly, improved OS was associated with high PNI relative to low PNI in the patients who accepted PD-L1/PD-1 inhibitors monotherapy or combined with chemotherapy (median OS: 18.5 vs. 11.0 months, HR = 0.33, *p* = 0.004 and median OS: 17.9 vs. 12.6 months, HR = 0.48, *p* = 0.005) (**Figures 2C, D**). The results revealed that an elevated PNI was associated with significantly lower risk of death in patients who accepted PD-L1/PD-1 inhibitors monotherapy or combined with chemotherapy (all *p* < 0.05).

### Association between the PNI and the predictive utility of SCLC patients received PD-L1/PD-1 inhibitors treatment

**Figure 3:**
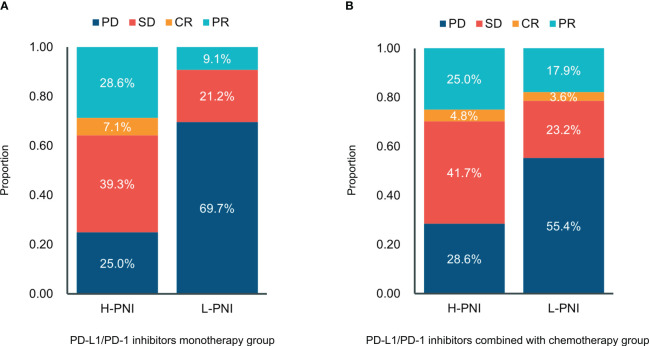
Distribution between responses and the PNI groups in patients with **(A)** PD-L1/PD-1 inhibitors monotherapy and **(B)** PD-L1/PD-1 inhibitors combined with chemotherapy. CR, complete response; PR, partial response; SD—stable disease; PD, progressive disease.

Of the 61 patients treated with PD-L1/PD-1 inhibitors monotherapy, 7 (25.0%) experienced progressive disease (PD), 11 (39.3%) experienced stable disease (SD), 8 (28.6%) experienced partial response (PR), and 2 (7.1%) experienced complete response (CR) in the High PNI group. This is compared with 23 (69.7%) patients with PD, 7 (21.2%) with SD, and 3 (9.1%) with PR in the low PNI group (**Figure 3A**). For patients received PD-L1/PD-1 inhibitors combined with chemotherapy, of the 84 patients in the High PNI group, 24 (28.6%) experienced PD, 35 (41.7%) experienced SD, 21 (25.0%) experienced PR, and 4 (4.8%) experienced CR in the High PNI group. Among the 56 patients in the Low PNI group, 31 (55.4%) experienced PD, 13 (23.2%) experienced SD, 10 (17.9%) experienced PR and 2 (3.6%) experienced CR (**Figure 3B**).

Moreover, the results of Point biserial correlation analysis between PNI and DCR showed that patients who had a higher increase in PNI trend had better DCR compared with those with a higher decrease in SCLC patients treated with PD-L1/PD-1 inhibitors monotherapy or combined with chemotherapy (r = 0.351, *p* < 0.001; r = 0.285, *p* < 0.001, respectively) (**Tables 2**, **3** **)**. In addition, compared with patients with high PNI, those with low PNI experienced worse ORR to PD-L1/PD-1 inhibitors monotherapy or combined with chemotherapy, although a benefit was noted, it was not statistically significant (r = 0.237, *p* =0.066; r = 0.106, *p =* 0.211, respectively) (**Tables 2**, **3**).

**Table 2:** Relationship between clinical response and PNI groups in SCLC patients treated with PD-L1/PD-1 inhibitors monotherapy.

Response	PNI Group [(n%)]		*p* value	Point biserial correlation coefficient PNI level	*p* value
	High PNI	Low PNI			
DCR			0.001	0.351	<0.001
CR+PR+SD	21(75.0)	10(30.3)			
PD	7(25.0)	23(69.7)			
ORR			0.011	0.237	0.066
CR+PR	10(35.7)	3(9.1)			
SD+PD	18(64.3)	30(90.9)			

PNI, prognostic nutritional index; SCLC, small cell lung cancer; CR, complete response; PR, partial response; SD, stable disease; PD, progressive disease; DCR, disease control rate; ORR, overall response rate.

**Table 3:** Relationship between clinical response and PNI groups in SCLC patients treated with PD-L1/PD-1 inhibitors combined with chemotherapy.

Response	PNI Group [(n%)]		*p* value	Point biserial correlation coefficient PNI level	*p* value
	High PNI	Low PNI			
DCR			0.001	0.285	<0.001
CR+PR+SD	60(71.4)	25(44.6)			
PD	24(28.6)	31(55.4)			
ORR			0.273	0.106	0.211
CR+PR	25(29.8)	12(21.4)			
SD+PD	59(70.2)	44(78.6)			

PNI, prognostic nutritional index; SCLC, small cell lung cancer; CR, complete response; PR, partial response; SD, stable disease; PD, progressive disease; DCR, disease control rate; ORR, overall response rate.

### Univariate and multivariate survival analyses of PFS and OS

**Figure 4:**
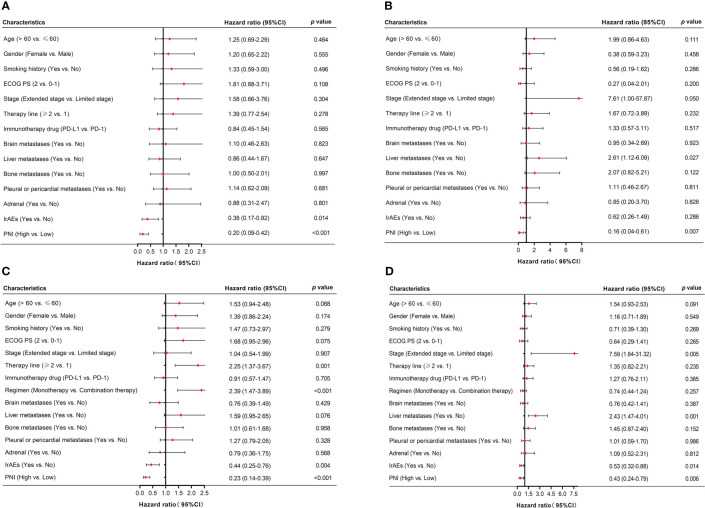
Univariate analysis of factors associated with PFS and OS in SCLC patients. **(A, B)** PFS and OS in SCLC patients treated with PD-L1/PD-1 inhibitors monotherapy. **(C, D)** PFS and OS in SCLC patients treated with PD-L1/PD-1 inhibitors combined with chemotherapy.

For patients received PD-L1/PD-1 inhibitors monotherapy, univariate Cox regression analysis showed that irAEs (*p* = 0.014) and PNI (*p* < 0.001) were significantly associated with PFS. Similarly, OS was associated with liver metastases (*p* = 0.027) and PNI (*p* = 0.007) (**Figures 4A, B** **)**. Moreover, in patients received PD-L1/PD-1 inhibitors combined with chemotherapy, univariate Cox regression analysis showed that therapy line (*p =* 0.001), regimen (*p <* 0.001), irAEs (*p* = 0.004), and PNI (*p* < 0.001) significantly affected PFS. In parallel, OS was significantly associated with stage (*p* = 0.005), liver metastases (*p* = 0.001), irAEs (*p* = 0.014), and PNI (*p =* 0.006) (**Figures 4C, D** **)**.

**Figure 5:**
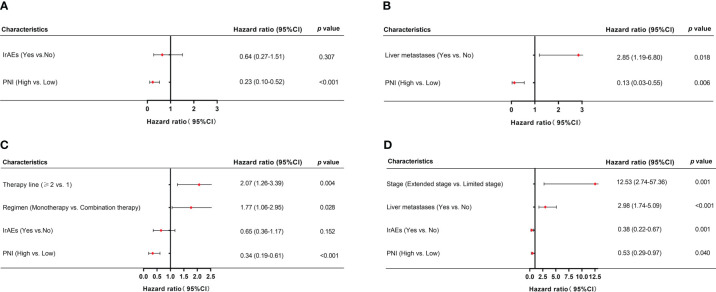
Multivariate analysis of factors associated with PFS and OS in SCLC patients. **(A, B)** PFS and OS in SCLC patients treated with PD-L1/PD-1 inhibitors monotherapy. **(C, D)** PFS and OS in SCLC patients treated with PD-L1/PD-1 inhibitors combined with chemotherapy.

Multivariate Cox-regression model showed that high PNI was significantly related to better PFS and OS in patients who accepted PD-L1/PD-1 inhibitors monotherapy or combined with chemotherapy (PD-L1/PD-1 inhibitors monotherapy: PFS: HR = 0.23, 95% CI: 0.10–0.52, *p* < 0.001 and OS: HR = 0.13, 95% CI: 0.03–0.55, *p =* 0.006; PD-L1/PD-1 inhibitors combined with chemotherapy: PFS: HR = 0.34, 95% CI: 0.19–0.61, *p* < 0.001 and OS: HR = 0.53, 95% CI: 0.29–0.97, *p =* 0.040, respectively) (**Figures 5A–D**). In addition, irAEs was also an independent prognostic factor for better OS in patients who accepted PD-L1/PD-1 inhibitors combined with chemotherapy (HR = 0.38, 95% CI: 0.22–0.67, *p =* 0.001) (**Figure 5D**). These results demonstrated that PNI was an independent prognostic factor for PFS and OS in patients who accepted PD-L1/PD-1 inhibitors or combined with chemotherapy.

### Immune-related adverse events (irAEs)

**Figure 6:**
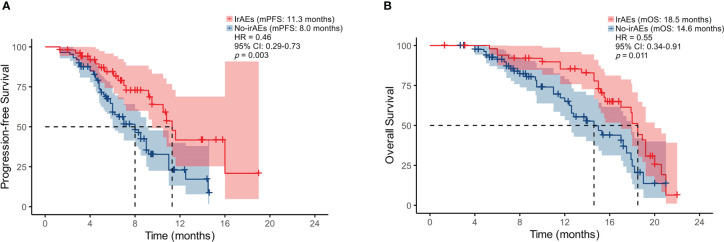
Kaplan-Meier analysis of PFS **(A)** and OS **(B)** based on the onset of irAEs.

In our study, 37.1% (n = 52) patients experienced six different irAEs of any grade. Among these, 25 (48.1%) experienced rash, 9 (17.3%) experienced hypothyroidism, 7 (13.5%) experienced liver dysfunction, 7 (13.5%) experienced infusion reaction, 1 (1.9%) experienced impaired glucose regulation, and 3 (5.8%) experienced diarrhea. The most common severe irAE (grade ≥ 3) was rash (11.5%, n = 6). The median PFS of the 52 patients with irAEs was significantly better than the 88 patients without irAEs (11.3 vs. 8.0 months, *p =* 0.003) (**Figure 6A**). Similarly, the median OS of the 52 patients with irAEs was significantly better than the 88 patients without irAEs (18.5 vs. 14.6 months, *p* = 0.011) (**Figure 6B**).

The High ALB, High LYM, and High PNI groups were composed of 24 (46.2%), 25 (48.1%), and 38 (45.2%) patients, respectively (**Table 4**). In univariate logistic regression analyses, the High LYM group and the High PNI group were significantly associated with any grade of irAEs (*p* = 0.041, *p* = 0.017). However, the PNI was not an independent prognostic risk factor of the onset of irAEs in the multivariate logistic regression analysis (*p* = 0.085).

**Table 4:** Levels of peripheral blood markers by irAEs development.

Blood markers	irAEs, n (%)	Univariate		Multivariate	
		OR (95% CI)	*p* value	OR (95% CI)	*p* value
L-ALB (n=88)	28 (31.8)	1			
H-ALB (n=52)	24 (46.2)	1.84 (0.91–3.72)	0.091		
L-LYM (n=88)	27 (30.7)	1			
H-LYM (n=52)	25 (48.1)	2.09 (1.03–4.25)	0.041	1.54 (0.70–3.38)	0.279
Low PNI (n=56)	14 (25.0)	1		1	
High PNI (n=84)	38 (45.2)	2.48 (1.18–5.20)	0.017	2.05 (0.90–4.65)	0.085

OR, odds ratio; CI, confidence interval; irAEs, immune-related adverse events; ALB, albumin; LYM, lymphocyte; PNI, prognostic nutritional index; L–ALB, Low–ALB; L–LYM, Low–LYM; H–ALB, High ALB; H–LYM, High LYM.

## Discussion

The emergence of PD-L1/PD-1 inhibitors has brought hope to patients with advanced SCLC, but the 5-year survival rate for patients remains low. Therefore, effective, reliable and easily accessible predictive biomarkers are urgently needed for identifying patients that will benefit from treatment. Currently, more focus has been turned toward the correlation between inflammatory-immune nutritional status and the clinical outcomes of cancer patients who are undergoing PD-L1/PD-1 inhibitor treatment. Systemic inflammation is closely associated with disease promotion and progression in most cancers, including lung cancer (23). PNI is obtained based on serum albumin and peripheral lymphocyte levels, which can reflect the nutritional and immune status of patients. In advanced head and neck cancers, a low PNI has been shown to positively correlate with worse survival and worse response rates to PD-L1/PD-1 inhibitors (19). However, whether the PNI can be used as a strong prognostic factor for SCLC patients has not yet been clarified. The aim of this study was to verify the predictive value of PNI for survival, treatment response rates, and treatment-related toxicity in SCLC patients of the China alpine region undergoing PD-L1/PD-1 inhibitor treatment.

In this retrospective study, the data were collected from the alpine region of China. The characteristics of the alpine region Chinese lung cancer population differ considerably from other lung cancer populations. The burden of lung cancer attributable to tobacco and PM 2.5 concentration in China alpine region remains heavy (24). As shown in **Table 1**, approximately 80% patients had a history of smoking. Our findings showed that low PNI was independently associated with worse PFS and OS in SCLC patients receiving PD-L1/PD-1 inhibitors monotherapy or combined with chemotherapy. Moreover, the correlation analysis showed that PNI status was positively correlated with DCR in SCLC patients receiving PD-L1/PD-1 inhibitors monotherapy or combined with chemotherapy (r = 0.351, *p* < 0.001; r = 0.285, *p* < 0.001, respectively). Johannet et al. also found that low PNI was associated with worse survival and treatment response rates in patients with liver cancer, melanoma, and uterine cancer (25). Immune-nutritional status prognosticates a response in SCLC patients treated with PD-L1/PD-1 inhibitors. Chronic inflammation associated with malnutrition inhibited adaptive immune system activation. Heightened levels of the proinflammatory cytokine interleukin-6 (IL-6) could induce endogenous steroid release and could further dampen immune cell functions, consequently reducing the effectiveness of PD-L1/PD-1 inhibitors (26, 27). Furthermore, T cells must acquire adequate nutrients to engage the metabolism which supports their functions. Metabolic competition between T cells and tumor cells in the tumor microenvironment leads to T cell hyporesponsiveness and further impairs PD-L1/PD-1 inhibitor efficacy (28, 29). Poor immune-nutritional status limits a response to PD-L1/PD-1 inhibitor treatment in SCLC patients and further leads to worse prognosis. The present analysis showed low PNI was significantly correlated with worse survival and a lower treatment response rate in SCLC patients treated with PD-L1/PD-1 inhibitors in the China alpine region population.

The occurrence of irAEs limits the use of ICIs. Therefore, early recognition and prompt intervention are particularly important. Wang et al. reported that 66% of patients undergoing PD-L1/PD-1 inhibitor monotherapy developed at least 1 irAEs of any grade in multiple solid tumor types (30). Our current research showed that patients with irAEs had better PFS and OS compared to those without irAEs (*p* = 0.003, *p* = 0.011), and these patients usually had a higher PNI status. Seiwert et al. also explored the association between the development of irAEs and prolonged OS in patients with head and neck cancer receiving ICIs (31). They demonstrated that ORR was higher for patients with irAEs compared to those without irAEs (30.6% vs. 12.3%, *p* = 0.020). Additionally, we explored an association between irAEs and peripheral blood markers and found that high PNI showed a trend towards being a prognostic factor for any grade of irAEs but did not reach the level of statistical significance (*p* = 0.085).

The present study demonstrated that pretreatment PNI is a promising efficacy and prognostic biomarker in SCLC patients treated with PD-L1/PD-1 inhibitors. Monitoring PNI status prior to PD-L1/PD-1 inhibitor treatment may significantly improve survival rate, current preventive and treatment approaches, and enhance accurate personal management of SCLC patients. Furthermore, the PNI can be easily calculated from peripheral blood counts, avoiding the need to obtain tumor specimens during the treatment process. Further prospective studies with larger sample sizes are necessary to confirm and support our conclusions.

In conclusion, we have demonstrated that low PNI was significantly correlated with worse survival and a lower treatment response rate, supporting its use as an effective biomarker in SCLC patients treated with PD-L1/PD-1 inhibitors. Improving nutrition and immune status by monitoring the PNI status of SCLC patients prior to PD-L1/PD-1 inhibitor treatment may optimize treatment efficacy and improve prognosis.

## Data availability statement

The raw data supporting the conclusions of this article will be made available by the authors, without undue reservation.

## Author contributions

Conceptualization and methodology: JYW, JY, YXQ, JYL, RZ, TL, LXL, and MW. Formal analysis, investigation, and data curation: JYW, TL, LXL, and MW. Writing—original draft preparation: JYW. Writing—review and editing: JYW, TL, LXL, and MW. Supervision and project administration: MW. All authors contributed to the article and approved the submitted version.
